# Covariate dependent Markov chains constructed with gradient boost modeling can effectively generate long-term predictions of obesity trends

**DOI:** 10.1186/s13104-023-06610-w

**Published:** 2023-11-24

**Authors:** Alexander A. Huang, Samuel Y. Huang

**Affiliations:** 1 Cornell University, Ithaca, NY USA; 2 Northwestern University Feinberg School of Medicine, Chicago, IL USA

**Keywords:** Machine-learning, Markov chains, Gradient boost modeling, Statistics, Mathematical modeling, Predictive modeling

## Abstract

**Importance:**

The prevalence of obesity among United States adults has increased from 30.5% in 1999 to 41.9% in 2020. However, despite the recognition of long-term weight gain as an important public health issue, there is a paucity of studies studying the long-term weight gain and building models for long-term projection.

**Methods:**

A retrospective, cross-sectional cohort study using the publicly available National Health and Nutrition Examination Survey (NHANES 2017–2020) was conducted in patients who completed the weight questionnaire and had accurate data for both weight at time of survey and weight ten years ago. Multistate gradient boost modeling classifiers were used to generate covariate dependent transition matrices and Markov chains were utilized for multistate modeling.

**Results:**

Of the 6146 patients that met the inclusion criteria, 3024 (49%) of patients were male and 3122 (51%) of patients were female. There were 2252 (37%) White patients, 1257 (20%) Hispanic patients, 1636 (37%) Black patients, and 739 (12%) Asian patients. The average BMI was 30.16 (SD = 7.15), the average weight was 83.67 kilos (SD = 22.04), and the average weight change was a 3.27 kg (SD = 14.97) increase in body weight (Fig. 1). A total of 2411 (39%) patients lost weight, and 3735 (61%) patients gained weight (Table 1). We observed that 87 (1%) of patients were underweight (BMI < 18.5), 2058 (33%) were normal weight (18.5 ≤ BMI < 25), 1376 (22%) were overweight (25 ≤ BMI < 30) and 2625 (43%) were obese (BMI > 30).

From analysis of the transitions between normal/underweight, overweight, and obese, we observed that after 10 years, of the patients who were underweight, 65% stayed underweight, 32% became normal weight, 2% became overweight, and 2% became obese. After 10 years, of the patients who were normal weight, 3% became underweight, 78% stayed normal weight, 17% became overweight, and 2% became obese. Of the patients who were overweight, 71% stayed overweight, 0% became underweight, 14% became normal weight, and 15% became obese. Of the patients who were obese, 84% stayed obese, 0% became underweight, 1% became normal weight, and 14% became overweight.

**Conclusions:**

United States adults are at risk of transitioning from normal weight to becoming overweight or obese. Covariate dependent Markov chains constructed with gradient boost modeling can effectively generate long-term predictions.

## Introduction

The prevalence of obesity among United States adults has increased from 30.5% in 1999 to 41.9% in 2020 [1–6]. Thus, new methods for modeling this increase in obesity overtime is necessary to best combat this public health emergency [7–10]. Multiple studies have identified major risk factors for obesity, including demographic factors (race, sex, age), lifestyle factors (exercise, sleep), and clinical comorbidities (hypertension, diabetes. Additionally, multiple studies have demonstrated the high prevalence of weight gain in the United States population and detailed the significant medical consequences of obesity related diseases such as coronary artery disease and type II diabetes [11–17]. Comorbidities such as cardiometabolic health and increased modeling of long-term diseases are of great focus in the building of models for obesity to guide medical care and limit the negative impacts of the obesity epidemic [18–21]. Despite the recognition of long-term weight gain as an important public health issue, there is a paucity of studies studying the long-term weight gain and building models for long-term projection [18, 20].

Markov chains are a very popular methodology to model change overtime, in which a transition probability is estimated, and the chain is executed overtime to generate long-term predictions [22–26]. Additionally, machine-learning techniques have been utilized to enhance predictive accuracy beyond that of parametric methods such as logistic and linear regression [27, 28]. Furthermore, very few studies utilize multiple states as their outcome, often choosing to rely on binary outcomes (obesity, not obesity), losing significant information in the process [29]. Development of a methodology to combine Markov chains, machine-learning techniques, and multi-state modeling will not only allow for accurate modeling of patient weight overtime, but also provide a new methodology that can be utilized to widely model any multi-state outcome, leading to broad scientific benefit [18, 20].

This study aims to provide a develop a methodology that utilizes multi-class machine learning classifiers with Markov chains to model obesity rates overtime in United States Adults within the National Health and Nutrition Examination Survey (NHANES) cohort [30–32].

## Methods

A retrospective, cross-sectional cohort study using the publicly available National Health and Nutrition Examination Survey (NHANES 2017–2020) was conducted in patients who completed the weight questionnaire and had accurate data for both weight at time of survey and weight ten years ago. The full dataset can be found on the cdc website (https://wwwn.cdc.gov/nchs/nhanes/continuousnhanes/default.aspx?Cycle=2017-2020) and a full detailed description of the questionaaire can be found.

### Ethics approval and consent to participate

The acquisition and analysis of the data within this study was approved by the National Center for Health Statistics Ethics Review Board.

### Dataset and cohort selection

The National Health and Nutrition Examination Survey (NHANES) is a program designed by the National Center for Health Statistics (NCHS), which has been leveraged to assess the health and nutritional status of the United States population. The NHANES dataset is a series of cross-sectional, complex, multi-stage surveys conducted by the Centers for Disease Control and Prevention (CDC) on a nationally representative cohort of the United States population to provide health, nutritional, and physical activity data. In the present study, we analyzed adult (≥ 18 years old) patients in the NHANES dataset if they completed the weight questionnaire, leading to the inclusion of 6146 total patients.

### Assessment of long-term weight change

From the questionnaire dataset in NHANES, the weight 10 years prior to the study and the current weight of the patient were extracted. The difference between the patient’s weight and the weight 10 years ago was the metric used for Long-term Weight Change within this study. Weight categories defined by the Centers of Disease Control and Prevention (CDC) were utilized in this study: Patients who had a BMI less than 18.5 were considered underweight, patients with BMI between 18.5 and 25 were considered of normal weight, patients with BMI between 25 and 30 were overweight, and patients with BMI above 30 were considered obese. These are in accordance to CDC categories and definitions did not vary by age ranges.

### Model construction and statistical analysis

Overall weight change was summarized within a histogram and the state transitions were summarized within in a transition-state figure. Additionally, patients were classified as underweight, normal weight, overweight, or obese at both the time of survey and ten years prior. A transition matrix was built based upon this cohort and Markov Chains were used to project which states (underweight, normal weight, overweight, or obese) the patient was likely to be in for the next 10 decades. Covariates were taken from NHANES dataset and focused upon race and sex. These projections were plotted visually in line-graphs. Confidence intervals for these estimates were obtained though bootstrap simulation of the transition matrices. Additionally, multistate gradient boost modeling classifiers were used to generate covariate dependent transition matrices and Markov chains were utilized for multistate modeling.

Gradient boost modeling methodology: Patients were split into four groups (underweight, normal weight, overweight and obese). A model was built for each of these groups, and the gradient boost modeling was utilized to predict the probability that any patient in the group would transition to the other states.

## Results

**Fig. 1:**
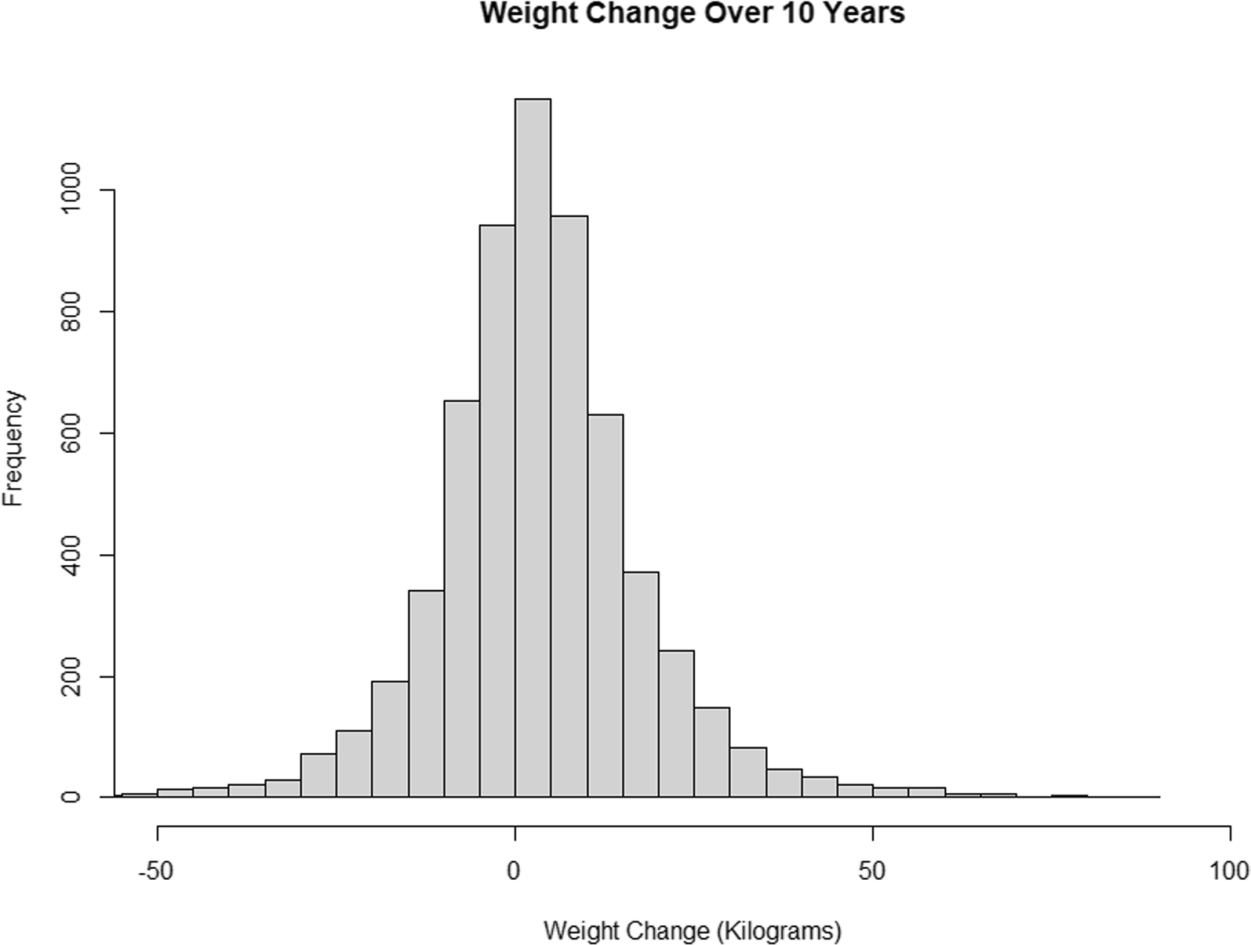
Histogram of 10-year weight change

Of the 6146 patients that met the inclusion criteria, 3024 (49%) of patients were male and 3122 (51%) of patients were female. There were 2252 (37%) White patients, 1257 (20%) Hispanic patients, 1636 (37%) Black patients, and 739 (12%) Asian patients (Table 1). The average BMI was 30.16 (SD = 7.15), the average weight was 83.67 kilos (SD = 22.04), and the average weight change was a 3.27 kg (SD = 14.97) increase in body weight (Fig. 1). A total of 2411 (39%) patients lost weight, and 3735 (61%) patients gained weight. We observed that 87 (1%) of patients were underweight (BMI < 18.5), 2058 (33%) were normal weight (18.5 ≤ BMI < 25), 1376 (22%) were overweight (25 ≤ BMI < 30) and 2625 (43%) were obese (BMI > 30).

**Table 1:** Summary of patient characteristics, stratified by patients with weight loss vs no weight loss

	All patients	Lost weight	Gained or maintained weight	P-value
Total patients (N)	6146 (1)	2411 (0.39)	3735 (0.61)	N/A
Age; mean (SD)	58.39 (12.94)	62.01 (12.85)	56.05 (12.45)	P < 0.001
Gender male; count (%)	3024 (0.49)	1376 (0.57)	1648 (0.44)	P < 0.001
Gender female; count (%)	3122 (0.51)	1035 (0.43)	2087 (0.56)	
Race white; count (%)	2252 (0.37)	943 (0.39)	1309 (0.35)	P < 0.001
Race other; count (%)	262 (0.04)	113 (0.05)	149 (0.04)	
Race hispanic; count (%)	1257 (0.2)	462 (0.19)	795 (0.21)	
Race black; count (%)	1636 (0.27)	609 (0.25)	1027 (0.27)	
Race asian; count (%)	739 (0.12)	284 (0.12)	455 (0.12)	
Income_poverty_ratio; mean (sd)	2.7 (1.63)	2.59 (1.61)	2.77 (1.64)	p = 0.53
BMXWT—weight (kg); mean (SD)	83.67 (22.04)	76.89 (18.94)	88.04 (22.79)	P < 0.001
weight_change; mean (SD)	3.27 (14.97)	− 9.71 (10.39)	11.64 (10.96)	P < 0.001
BMXBMI—body mass index (kg/m**2); Mean (SD)	30.16 (7.15)	27.58 (5.96)	31.82 (7.36)	P < 0.001
Direct HDL-cholesterol (mg/dL); Mean (SD)	53.99 (16.34)	56.22 (17.36)	52.56 (15.48)	P < 0.001
LDL-cholesterol, friedewald (mg/dL); Mean (SD)	110.89 (37.05)	105.63 (36.74)	114.41 (36.84)	P < 0.001
Cholesterol, refrigerated serum (mg/dL); Mean (SD)	189.64 (41.88)	184.65 (43.57)	192.86 (40.44)	P < 0.001
Triglyceride (mg/dL); mean (SD)	114.94 (97.1)	105.21 (76.42)	121.43 (108.26)	P < 0.001
Albumin, urine (mg/L); mean (SD)	60.16 (367.89)	82.35 (490.35)	45.95 (259.97)	P < 0.001
Creatinine, urine (mg/dL); mean (SD)	121.99 (80.62)	116.61 (77.26)	125.44 (82.52)	P < 0.001
Albumin creatinine ratio (mg/g); mean (SD)	61.17 (396.2)	83.55 (517.76)	46.85 (292.24)	P < 0.001
Total cholesterol (mg/dL); mean (SD)	189.33 (41.85)	184.25 (43.52)	192.6 (40.41)	P < 0.001
Platelet count (1000 cells/uL); mean (SD)	241.76 (65.7)	234.72 (67.21)	246.32 (64.3)	P < 0.001
Insulin (pmol/L); mean (SD)	90.79 (150.55)	75.21 (129.96)	101.13 (161.99)	P < 0.001
Iron frozen, serum (ug/dL); mean (SD)	85.5 (34.49)	86.85 (35.02)	84.62 (34.12)	P < 0.001
Fasting glucose (mg/dL); mean (SD)	117.65 (40.76)	121.14 (46.42)	115.32 (36.34)	P < 0.001
Albumin, refrigerated serum (g/dL); mean (SD)	4.03 (0.33)	4.04 (0.34)	4.02 (0.32)	P < 0.001
Glucose, refrigerated serum (mg/dL); mean (SD)	106.12 (40.39)	110 (48.29)	103.61 (34.14)	P < 0.001
Phosphorus (mg/dL); mean (SD)	3.55 (0.53)	3.58 (0.55)	3.52 (0.51)	P < 0.001
Uric acid (mg/dL); mean (SD)	5.48 (1.48)	5.39 (1.49)	5.54 (1.48)	P < 0.001
SMDANY—used any tobacco product last 5 days?; Mean (SD)	1249 (0.2)	565 (0.23)	684 (0.18)	P < 0.001

**Fig. 2:**
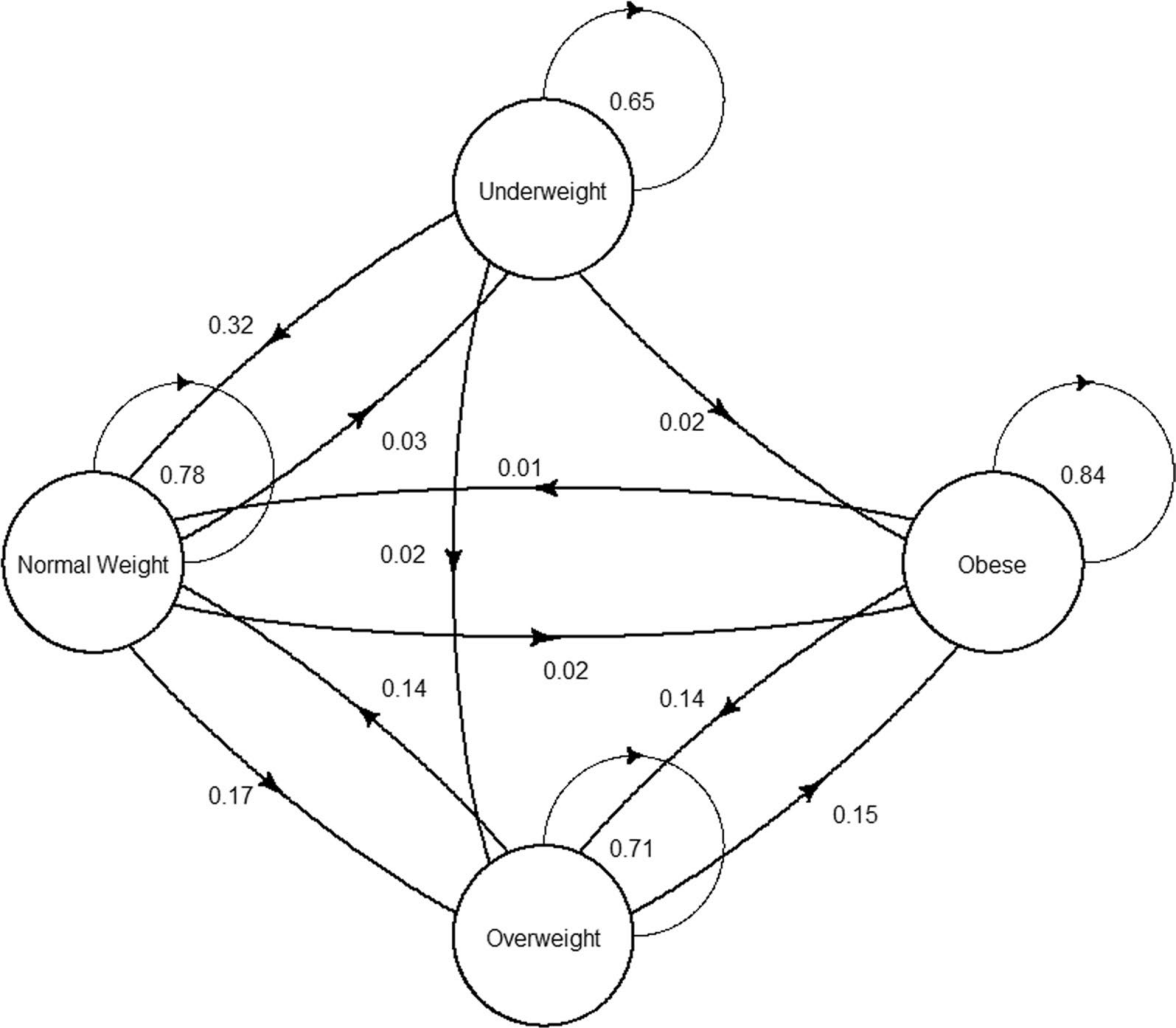
Transitions between obese, overweight, normal weight, and underweight. Each circle represents a specific weight category, and arrows represent the proportion of one group transitioning to another group over the 10-year period

**Fig. 3:**
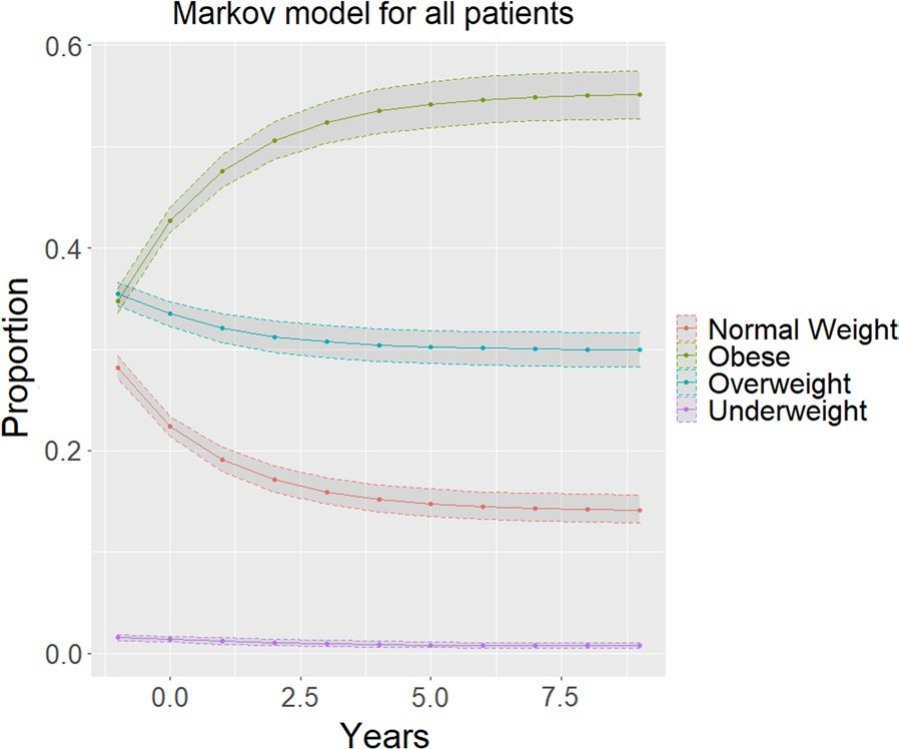
Markov model of weight overtime—each new dot represents a decade. X-axis years in units of 10 years. Y-axis is the prevalence of each weight category. Time prior to zero is modeling backwards for increased context for long-term projection

From analysis of the transitions between normal/underweight, overweight, and obese, we observed that after 10 years, of the patients who were underweight, 65% stayed underweight, 32% became normal weight, 2% became overweight, and 2% became obese (Figs. 2, 3). Full 10-year transition trajectories present in Fig. 2. After 10 years, of the patients who were normal weight, 3% became underweight, 78% stayed normal weight, 17% became overweight, and 2% became obese. Of the patients who were overweight, 71% stayed overweight, 0% became underweight, 14% became normal weight, and 15% became obese. Of the patients who were obese, 84% stayed obese, 0% became underweight, 1% became normal weight, and 14% became overweight (Fig. 2).

Subgroup analyses allow for visualization of potential covariates. Use of Gradient boost modeling shows statistically identical distributions for the Markov projection overtime, thus validating this methodology. Furthermore, gradient boost modeling was able to be used for continuous covariates, an example was computed with a three covariate model: age, race, and gender. An example for Age = 35, Race = Black, and Gender = Female was computed, and an example for age = 65, Race = Asian, and Gender = Male was computed to demonstrate the utility of the covariate dependent Markov chain in modeling change overtime.

## Discussion

**Fig. 4:**
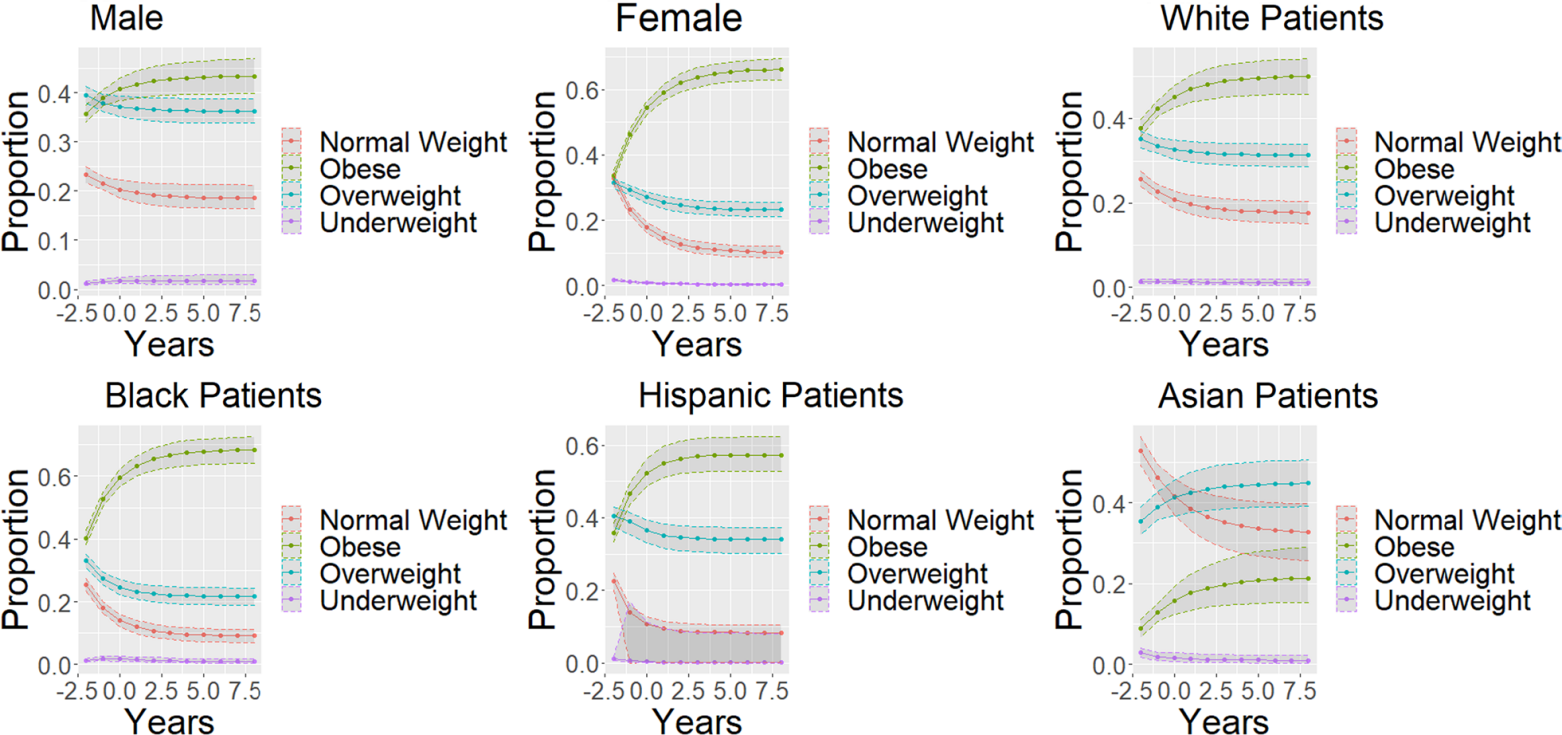
Sex and weight differences in weight overtime—each new dot represents a decade. X-axis years in units of 10 years. Y-axis is the prevalence of each weight category

In this retrospective, cross sectional cohort of United States adults, we observed that patients on average gained 3.27 kg over the 10 year period. As a result of this age-related weight gain, we observed that a large number of patients who were normal weight transitioning to become overweight or obese within a decade. On subgroup analysis, we found that female patients were more likely to gain weight than male patients (Fig. 4). Furthermore, we found that compared wo white patients, Black and Hispanic patients were more likely to gain weight and Asian patients were less likely to gain weight (Fig. 4). These observed differences highlights that there may be additional demographic, lifestyle, and biological risk factors for obesity that should be analyzed. Many of these risk factors have been examined in the literature, including increased intake of sugar, decreased exercise, increased sedentary activity, and decreased sleep [11, 13, 14, 16, 33].

**Fig. 5:**
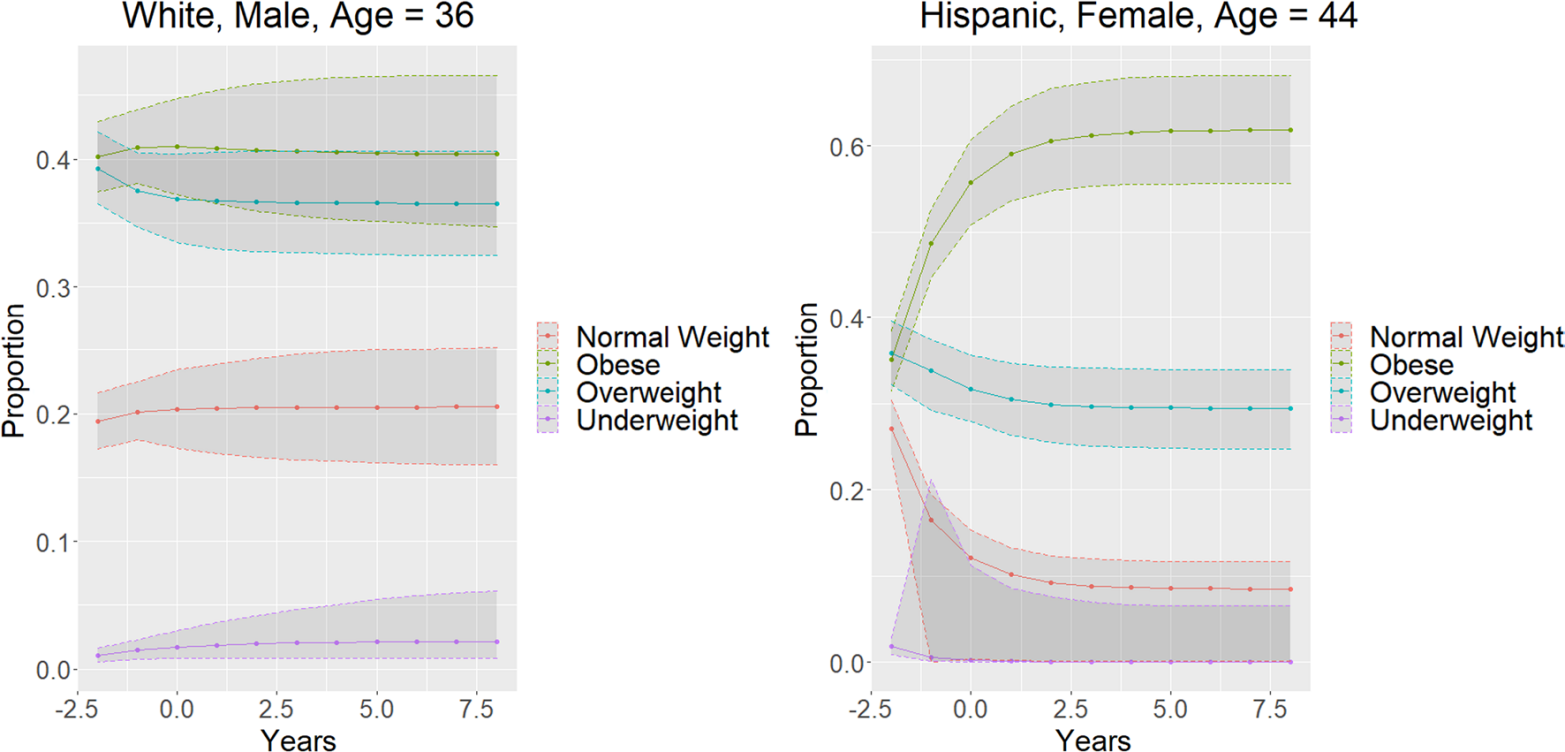
Modeling of two particular patients based upon race, sex, and age—each new dot represents a decade. X-axis years in units of 10 years. Y-axis is the prevalence of each weight category

Recognizing that there are numerous clinical and demographic covariates that may help model weight change overtime, a clear methodology that is able to help model weight change over time accurately is needed [3, 12]. Our methodology combines multistate modeling with Markov chains with machine-learning techniques. Multistate modeling allows us to model obesity as a set of four outcomes: underweight, normal weight, overweight, and obese. Other methods used to model obesity use logistic regression, in which the model is limited to a binary outcome, often obesity vs no obesity. By utilizing multistate modeling, a more accurate depiction of a patient’s weight can be done, and no loss of information is present. Furthermore, using multistate modeling through Markov chains allows for extrapolation into the future. Another innovation is the combination of machine learning with Markov chains for multistate modeling. Most methods that use machine learning to model outcomes often model binary outcomes (obesity vs no obesity). However, we were able to utilize multi-class classification instead of binary classifiers and be able to have the machine learning have output of underweight, normal weight, overweight, and obese instead of traditional binary outcomes of obese vs not obese. By being able to combine the multi-class classification of machine learning models, (in our case gradient boost modeling) combined with Markov chains, we are able to have the utility for long-term projection Markov chains offer as well as the predictive accuracy that machine learning algorithms are able to offer (Fig. 5).

What our study contributes to the literature is a novel method for modeling obesity trends over time. We are able to effectively combine two well validated techniques for predictive modeling: Markov chains and supervised machine learning (gradient boost modeling). Additionally, the novel use of bootstrap simulations allowed for accurate quantification of the uncertainty of each of these observations that were constructed by the Markov chains [34–36]. This novel method of applying covariate-dependent transition states within Markov chains has utility beyond obesity metrics. Any multistate outcome of interest can be modeled, and the transition states being dependent on any characteristics of the patient can be completed using this methodology. Furthermore, we were able to validate the reliability of this methodology by comparing univariable models with subgroups. The near identical match of the predictions from our algorithm and the subgroups gives confidence that this methodology is able to generate unbiased estimations.

### Limitations

This study has several strengths and weaknesses. We utilized the NHANES dataset, which is a retrospective cohort, carrying the limitations of retrospective studies. However, this study allows for the selection of a large cohort, evaluation of data quality, and due to the publicly available nature of the cohort, allows for increased replication and follow-up studies based upon the same cohort. Furthermore, the cohort relied on surveys to obtain the outcome of interest (weight 10 years ago) as well as the dietary and lifestyle information. More accurate measurements may have been achieved with prospective studies with lab measurements of weight, but these may interfere with natural patient habits since they know they are part of a nutrition study and may have significantly different behaviors than the general population. Additionally, self-reported survey information allows for the volume of participants to be included within this study. Another weakness was the voluntary nature of this cohort, with participants choosing to opt into the study instead of being randomly selected. This may artificially select a different cohort that may significantly differ from the population. However, our analysis found a demographically diverse population, so these results may still be generalizable to other cohorts.

### Conclusion

United States adults are at risk of transitioning from normal weight to becoming overweight or obese. Covariate dependent Markov chains constructed with gradient boost modeling can effectively generate long-term predictions.

## Data Availability

The data from this cohort can be found on the NHANES section of the CDC website. https://wwwn.cdc.gov/nchs/nhanes/continuousnhanes/default.aspx?cycle=2017-2020.
